# TGF-β inhibits IL-1β-activated PAR-2 expression through multiple pathways in human primary synovial cells

**DOI:** 10.1186/1423-0127-16-97

**Published:** 2009-10-23

**Authors:** Shin-Han Tsai, Ming-Thau Sheu, Yu-Chih Liang, Hsiu-Tan Cheng, Sheng-Shiung Fang, Chien-Ho Chen

**Affiliations:** 1 Department of Neurosurgery, Department of Emergency and Critical Care Medicine, Taipei Medical University-Shuang Ho Hospital, Taipei, Taiwan, Republic of China; 2 Graduate Institute of Pharmaceutical Sciences, Taipei Medical University, Taipei, Taiwan, Republic of China; 3 School of Medical Laboratory Science & Biotechnology, Taipei Medical University, Taipei, Taiwan, Republic of China; 4 Department of Laboratory Medicine, Taipei Medical University Hospital, Taipei, Taiwan, Republic of China

## Abstract

To investigate the mechanism how Transforming growth factor-β(TGF-β) represses Interleukin-1β (IL-1β)-induced Proteinase-Activated Receptor-2 (PAR-2) expression in human primary synovial cells (*h*PSCs). Human chondrocytes and *h*PSCs isolated from cartilages and synovium of Osteoarthritis (OA) patients were cultured with 10% fetal bovine serum media or serum free media before treatment with IL-1β, TGF-β1, or Connective tissue growth factor (CTGF). The expression of PAR-2 was detected using reverse transcriptase-polymerase chain reaction (RT-PCR) and western blotting. Collagen zymography was performed to assess the activity of Matrix metalloproteinases-13 (MMP-13). It was demonstrated that IL-1β induces PAR-2 expression via p38 pathway in *h*PSCs. This induction can be repressed by TGF-β and was observed to persist for at least 48 hrs, suggesting that TGF-β inhibits PAR-2 expression through multiple pathways. First of all, TGF-β was able to inhibit PAR-2 activity by inhibiting IL-1β-induced p38 signal transduction and secondly the inhibition was also indirectly due to MMP-13 inactivation. Finally, TGF-β was able to induce CTGF, and in turn CTGF represses PAR-2 expression by inhibiting IL-1β-induced phospho-p38 level. TGF-β could prevent OA from progression with the anabolic ability to induce CTGF production to maintain extracellular matrix (ECM) integrity and to down regulate PAR-2 expression, and the anti-catabolic ability to induce Tissue inhibitors of metalloproteinase-3 (TIMP-3) production to inhibit MMPs leading to avoid PAR-2 over-expression. Because IL-1β-induced PAR-2 expressed in *h*PSCs might play a significantly important role in early phase of OA, PAR-2 repression by exogenous TGF-β or other agents might be an ideal therapeutic target to prevent OA from progression.

## Background

Osteoarthritis (OA) is a degenerative disease characterized by depletion of articular cartilage and formation of osteophytes [1]. OA formed under the condition of imbalance between anabolic and catabolic mediators, when catabolism is greater than anabolism, the risk of OA raises. The catabolic mediators include MMPs, ADAMTS, ADAM, IL-1β, IL-17, IL-18 and TNF-α, which increase degradation of cartilage and inhibit synthesis of metalloproteinase inhibitors such as TIMPs, tenascin and YKL-40. The anabolic mediators include TGF-β, IGF-1, FGFs and BMPs, which stimulate synthesis and repairing of cartilage. The secreted proinflammatory cytokines and metalloproteinases up-regulate expression of chondrocyte PAR-2, stimulating more secretion of proinflammatory cytokines and metalloproteinases to enhance inflammatory response [2,3] and degradation of ECM components of cartilage tissue, causing progressive loss of cartilage. Furthermore, fragments of protein degradation, like fibronectin fragments and collagen type II fragments, seem to play a role in inducing degradation of cartilage [4,5] as well as stimulating chondrocytes to repair the matrix.

TGF-β, a prominent member of the TGF-β superfamily of ligands which include TGF-β s and BMPs, is vital for the homeostasis of numerous cellular functions, including cell growth, differentiation, and apoptosis in a broad spectrum of tissues [6]. TGF-β signals are propagated through direct physical interactions with the extracellular domain of essentially two transmembrane serine/threonine kinase receptors (Tβ-RI and Tβ-RII), which transduce a number of secondary signals, most notably Smads 2 and 3 as well as PI3-kinase and various members of the mitogen activated protein kinase (MAPK) family [7-10]. CTGF, a member of CCN family, is a cysteine-rich matricellular protein. Expression of this protein is potently induced by TGF-β via Smad pathway. CTGF promotes chondrocytes proliferation through p38 MAPK and differentiation via p42/p44 MAPK. Thus, CTGF is important for cell proliferation and matrix remodeling during chondrogenesis and is a key regulator coupling ECM remodeling [11]. Several studies have proved that CTGF can stimulate the proliferation and expression of the cartilage phenotype by promoting type II collagen and aggrecan production, but did not stimulate the terminal hypertrophy or calcification of articular cartilage cells, suggesting that CTGF might be useful in the repair of damaged articular cartilage [12-14]. Other report suggested that TGF-β antagonizes IL-1β-mediated inflammation via decreasing its receptor expression on chondrocytes [15-17] and TGF-β and CTGF play a critical role in cartilage matrix repairing; in addition, TGF-β is an anabolic and anti-catabolic factor of articular cartilage.

PARs are a family of four G-protein-coupled receptors which included four members: PAR-1, PAR-2, PAR-3, and PAR-4 [18]. Among PARs, PAR-2 is unique in that it is activated by trypsin and mast cell tryptase, but not by thrombin which activates the other three members of the PAR family. This study focuses on PAR-2, which plays an important role in inflammation and pain. Trypsin cleaves PAR-2 at R^34^↓S^35^LIGKV of the extracellular N-terminus to expose the hexameric tethered peptide that binds to conserved regions in extracellular second loop of the receptor to initiate signaling. During activation, PAR-2 couples to Gα q/11, resulting in activation of phospholipase C-β, production of inositol 1,4,5-trisphosphate and diacylglycerol, and then activation of protein kinase C. In addition, PAR-2 can activate ERK1/2 MAPK, mediating cell proliferation [11]. Recently, it was reported that PAR-2 was expressed on chondrocytes and synovial cells and it was overexpressed on osteoarthritic chondrocytes. The expression level of PAR-2 on chondrocytes is up-regulated by IL-1β and TNF-α but down-regulated by TGF-β[12].

In human chondrocytes, a recent study has reported that TGF-β induced TIMP-3 via PI3K/Akt signaling pathway [19]. Several models have been proposed to explain how TGF-β may activate the PI3K/Akt pathway. A recent study further suggests that Tβ-RI associates with the p85 regulatory subunit of PI3K, thus enabling activation of the p110α catalytic subunit of PI3K [6]. In this study, we aim to study the mechanism how TGF-β represses IL-1β-induced PAR-2 expression in human primary synovial cells (*h*PSCs). Our findings emphasize that TGF-β represses IL-1β-induced PAR-2 expression via multiple pathways including TGF-β directly inhibits IL-1β induced p-p38 level; TGF-β represses human synovial PAR-2 via Akt-TIMP3-MMP13 pathway; TGF-β induces CTGF formation, and then CTGF reduces PAR-2 protein level in *h*PSCs.

## Materials and methods

### Isolation and culture of human primary chondrocytes and synovial cells (*h*PSC)

Joint tissues were obtained from patients with OA undergoing implant surgery for total knee replacement. Articular tissues was surgically removed and separated from adjacent non-lesional tissues that appeared to be morphologically normal. Chondrocytes and synovial cells from those specimens were isolated as described. Briefly, tissues specimens were first cut into pieces (2~3 mm^3^), and chondrocytes and synovial cells were released from the articular tissues by sequential incubation with 1 mg/mL hyaluronidase (Sigma Chemical St. Louis, Mo, USA) for 15 min, 0.25% proteinase for 30 min, and 2 mg/mL type II collagenase (Sigma) for 12 h at 37°C in Dulbecco's modified Eagle medium (DMEM) (Gibco BRL, Life Technologies, Grand Island, NY, USA). After isolation, chondrocytes and synovial cells were resuspended in DMEM containing 10% FBS and 1% L-glutamine, and then incubated at 37°C in a humidified atmosphere with 5% CO_2 _and 95% air. All experiments of cell starvation were intended to render quiescent.

### Reverse-transcription polymerase chain reaction (RT-PCR)

The extracted RNA (2 μg) was reverse-transcribed at 37°C for 1.5 h by adding 5 μM of random hexamer oligonucleotides (Gibco BRL, Life Technologies, Grand Island, NY, USA), 200 units of reverse transcriptase (Takara Bio Inc., Japan), 2.5 mM deoxyribonucleotide triphosphate (dNTP) (Takara Bio.), and 10 mM dithiothreitol. PCR primers for amplification of MMP-1, MMP-3, MMP-13, IL-8, and GAPDH cDNA were synthesized according to the following oligonucleotide sequences. **CTGF**: Sense, 5' CCG TAC TCC CAA AAT CTC CA, Anti-sense, 5' GTA ATG GCA GGC ACA GGT CT; **PAR-1**: Sense, 5'ACC ACA TTT GCT CCA TCC TC, Anti-sense, 5' CCT ATT GGA GTG CCC ACA GT; **PAR-2**: Sense, 5' CTG CCT ATG TGC TGA T, Anti-sense, 5' CGG ACA CTT CGG CAA A; **PAR-3**: Sense, 5'ACC CTC CAC CAC TTC ACA AG, Anti-sense, 5'AGC AAG AGG TTT GGT TGG TG; **PAR-4**: Sense, 5'GTG GGC CTT ACA TCC AGT GT, Anti-sense, 5'CCT TCT GCC TCA GTC TCC TG; **TIMP-3**: Sense, 5'CCT TCT GCC TCA GTC TCC TG, Anti-sense, 5' AAG AAG CCT CTA CCC CCA AA; **GAPDH**: Sense, 5'CAA GGC TGA GAA CGG GAA GC, Anti-sense, 5'AGG GGG CAG AGA TGA TGA CC. PCR was carried out with 2 μl of template cDNA and 23 μl of PCR mix buffer containing each primer (0.2 μM), dNTP (2.5 mM), and Taq DNA polymerase (1.25 units) (Takara Bio.). After the PCR, 15 μl of the reaction mixture was subjected to electrophoresis on a 1.5% agarose gel, and the PCR products were visualized by ethidium bromide staining. The levels of mRNA for MMPs and GAPDH were quantified by scanning densitometry (Image-Pro Plus, Media Cybernetics, MD, USA). Equal loading of DNA samples in each lane was indicated by equal intensity of internal control, GAPDH. The data shown here are representative of three independent experiments.

### Western blot analysis

Cells were extracted from the total protein using ice-cold RIPA lysis buffer (10 mM Tris-HCl (pH 7.6), 158 mM NaCl, 1.0 mM EDTA, 0.1% SDS, 1% Triton X-100, 1.0 mg/ml leupeptin, 1.0 mg/ml aprotinin, and 0.5 mM PMSF). The lysates were transferred to Eppendorf tubes and centrifuged at 14,000 rpm for 30 min at 4°C. The supernatants were transferred to fresh tubes, and the protein concentration was determined using the Bio-Rad Protein assay. Similar amounts of protein were separated by 10% SDS-PAGE and transferred to a nitrocellulose membrane (Gelman, Ann Arbor, MI, USA) by electroblotting. The membrane was blocked overnight in a 5% milk powder/TBST solution and then further incubated with one of the following antibodies (Abs): MMP-1, -3, or -13 Ab (Oncogene, Merck, Taipei, Taiwan) for 2 h. Membranes were washed three times with TBST, then further incubated with the appropriate HRP-labeled secondary Ab in 5% milk powder/TBST, and developed using an ECL system (Santa Cruz Biotechnology, Santa Cruz, CA, USA). Equal loading of protein samples in each lane was indicated by equal intensity of loading control protein, GAPDH. The data shown here are representative of three independent experiments.

### Collagen zymography

To analyze MMP-13 activities, concentrated media were mixed with sample buffer without reducing agent or boiling. The sample was loaded into 0.5 mg/ml collagen-containing SDS-polyacrylamide gel, and then undergoing electrophoresis. After electrophoresis, remove SDS from the gel by washing in 2.5% Triton X-100 solution for 30 minutes at room temperature that allows enzymes to reactivate and degrade the protein substrate. Then, the gel was rinsed with developing buffer containing 25 mM Tris-HCl, 0.2 M NaCl, 6 mM CaCl_2_, and 0.02% Brij for 30 minutes at room temperature and then incubation with developing buffer at 37°C overnight. Afterward, zymographic activities were revealed by staining with 0.5% CBB R-250 (Sigma) for 30 minutes at room temperature and destaining with CBB destainer.

### Statistical analysis

Results were normalized to the copy numbers of GAPDH or α-tubulin. The mean and standard deviation were used to evaluate the mRNA and protein level of CTGF, PAR-2, and TIMP-3. Student's *t *test was used for comparison of the difference in expression level of target proteins and target gene between samples within the same set. The effects of the first stimulation were done as positive control and were analyzed as changes relative to un-stimulated baseline, using repeated measure analysis of variance, and the inhibitory effects of the second drug co-treated with the first stimulant were analyzed as relative to positive control by using repeated measurement of variance. These analyses were performed individually and for the group as a whole. Statistical significance was set as *: *P *< 0.05 and **: *P *< 0.01 comparing to basal control and $: *P *< 0.05 and $$: *P *< 0.01 comparing to positive control.

## Results

### Induction of PAR-1, PAR-2, PAR-3, and PAR-4 mRNA level by IL-1β in *h*PSCs and chondrocytes

**Figure 1:**
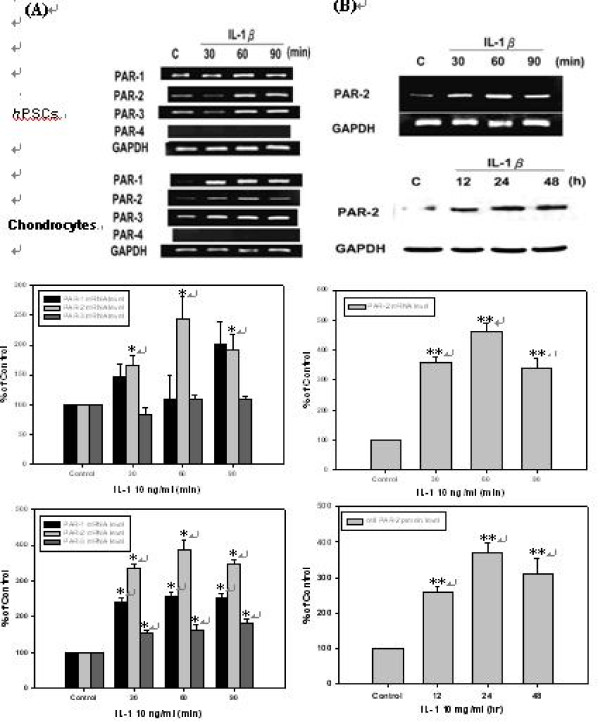
**Induction of PAR-1, PAR-2, PAR-3, and PAR-4 mRNA level by IL-1β in *h*PSCs and chondrocytes and Induction of PAR-2 mRNA and protein level by IL-1β in *h*PSCs**. **(A)**. *h*PSCs and chondrocytes were incubated in 10 ng/ml of IL-1β in serum-free DMEM for 30, 60, and 90 minutes following 24 hours of starvation and PAR-1, PAR-2, PAR-3, and PAR-4 mRNA level of ***h*PSCs **and chondrocytes were determined by RT-PCR (*n *= 3). Lane 1 as basal control and lane 2 to lane 4 were compared with basal control. **(B)**. *h*PSCs were incubated in 10 ng/ml of IL-1β in serum-free DMEM for 30, 60, and 90 minutes following 24 hours of starvation (*n *= 3). *h*PSCs were incubated with 10 ng/ml of IL-1β for 12, 24, and 48 hours (*n *= 3). Lane 1 as basal control and lane 2 to lane 4 were compared with basal control.

*h*PSCs and chondrocytes were incubated with 10 ng/ml of IL-1β in serum-free DMEM for 30, 60, and 90 minutes following 24 hours of starvation. Results shown by Fig. 1A demonstrates that the mRNA levels of PAR-1, PAR-2, and PAR-3 were significantly induced by IL-1β in human chondrocytes, but only PAR-2 mRNA was up-regulated by IL-1β in human primary synovial cell. In addition, PAR-2 had higher expression level in *h*PSCs than that in human chondrocytes, which suggests that PAR-2 in *h*PSCs might play a more significant role in the progression of OA.

### Induction of PAR-2 mRNA and protein level by IL-1β in *h*PSCs

For inducing PAR-2 mRNA expression, *h*PSCs were treated with 10 ng/ml of IL-1β for 30, 60, and 90 minutes, while PAR-2 protein level after treatment with 10 ng/ml of IL-1β was determined at 12, 24, and 48 hrs. As shown by Fig. 1B, IL-1 β induced mRNA expression of PAR-2 in *h*PSCs started at 30 minute and reached maximal effect at the time point of 1 hr (Fig. 1B, mRNA level). IL-1 β induced PAR-2 protein expression at 12 hr that elevated up to 48 hr (Fig. 1B, protein level).

### Inhibition of IL-1β-induced PAR-2 expression by p38 MAPK inhibitor but not by MEK or JNK MAPK inhibitor in *h*PSCs

**Figure 2:**
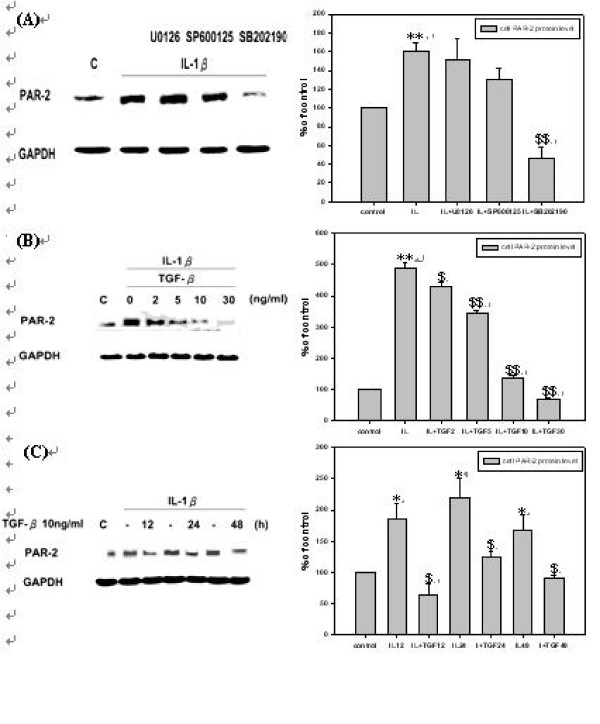
**Inhibition of IL-1β-induced PAR-2 expression by p38 MAPK inhibitor but not by MEK or JNK MAPK inhibitor and TGF-β1 suppresses PAR-2 protein level induced by IL-1β in *h*PSCs**. **(A) ***h*PSCs were pre-treated with 5 μM of U0126, SP600125, and SB202190 in serum-free DMEM for 1 hour and then incubated with 10 ng/ml of IL-1β for 12 hours in serum-free DMEM (*n *= 3). Lane 1 as basal control and lane 2 as positive control, lane 3 to lane 5 were compared with positive control. **(B) ***h*PSCs were pre-treated with 10 ng/ml of IL-1β in serum-free DMEM for 30 minutes and then incubated with 2, 5, 10, and 30 ng/ml of TGF-β1 in serum-free DMEM for 12 hours (*n *= 3). Lane 1 as basal control and lane 2 as positive control, lane 3 to lane 6 were compared with basal control. **(C) ***h*PSCs were pre-treated with 10 ng/ml of IL-1β in serum-free DMEM for 30 minutes and then incubated with 10 ng/ml of TGF-β1 for 12, 24, and 48 hours in serum-free DMEM (*n *= 3). Lane 1 as basal control and lane 2, 4, and 6 as positive controls of lane 3, 5, and 7. Lane 2, 4, and 6, were compared with basal control; lane 3 was compared with lane 2; Lane 5 was compared with lane 4; and lane 7 was compared with lane 6.

In order to elucidate the pathways by which *h*PSCs regulate PAR-2 level by IL-1β, MAPK inhibitors were pre-treated for 1 hr followed by stimulating with IL-1β for 12 hrs, and PAR-2 protein level was determined. As shown by Fig. 2A, SB202190, a p38 inhibitor, significantly inhibited IL-1β induced PAR-2 expression of protein level which was not inhibited by U0126, a MEK inhibitor or SP600125, a JNK inhibitor.

### TGF-β1 suppresses excess expression PAR-2 induced by IL-1β in *h*PSCs

According to a recent report, TGF-β can decrease over-expressed PAR-2 protein levels of chondrocytes from OA patients, but this phenomenon has not been observed in chondrocytes from non-OA patients that expressed normal PAR-2 level [2]. These results suggest that TGF-β is a regulator of PAR-2 expression in human chondrocytes. In light of this, the *h*PSCs were pre-treated with IL-1β for 30 minutes to mimic early OA condition to increase PAR-2 protein level, and then co-treated with various concentrations of TGF-β1 for 12 hrs or co-treated with 10 ng/ml of TGF-β1 for 12, 24, and 48 hrs. As shown by Fig. 2B and 2C, increasing PAR-2 protein level induced by IL-1β could be down-regulated by TGF-β1 in a dose-dependent (Fig. 2B) and a time-dependent manner from 12 to 48 hrs (Fig. 2C).

### TGF-β1 inhibits IL-1β mediated p38 MAPK activation, but not JNK or ERK level of *h*PSCs

**Figure 3:**
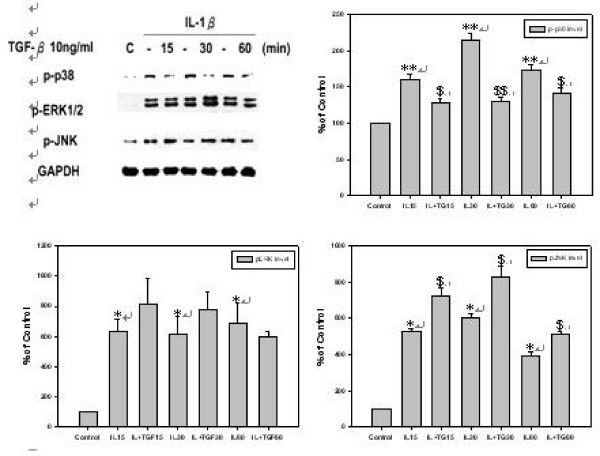
**TGF-β1 inhibits IL-1β-mediated p38 MAPK activation, but not JNK or ERK level**. *h*PSCs were first incubated in serum-free DMEM for 24 hours and then pre-treated with 10 ng/ml of IL-1β in serum-free DMEM for 30 minutes and then incubated with 10 ng/ml of TGF-β1 for 15, 30, and 60 minutes in serum-free DMEM (*n *= 3). Lane 1 as basal control and lane 2, 4, and 6 as positive controls of lane 3, 5, and 7. Lane 2, 4, and 6, were compared with basal control; lane 3 was compared with lane 2; Lane 5 was compared with lane 4; and lane 7 was compared with lane 6.

It was demonstrated as above that IL-1β induced PAR-2 expression of *h*PSCs via p38 MAPK pathway and TGF-β1 could reduce this induction of PAR-2 expression. Since that, the mechanisms by which TGF-β1 inhibits IL-1β-mediated PAR-2 expression in *h*PSCs were investigated. Cells were pre-treated with IL-1β for 30 minutes to mimic OA condition, followed by co-treating with TGF-β1 for various time periods from 15 to 60 minutes and then p-p38, p-ERK, and p-JNK level were analyzed. As shown by Fig. 3, TGF-β1 reduced IL-1β mediated p-p38 level, at the time points of 15, 30, and 60 minutes, but TGF-β1 had no inhibition effect on p-ERK or p-JNK levels.

### TGF-β1 induces mRNA and protein expression level of CTGF in *h*PSCs

**Figure 4:**
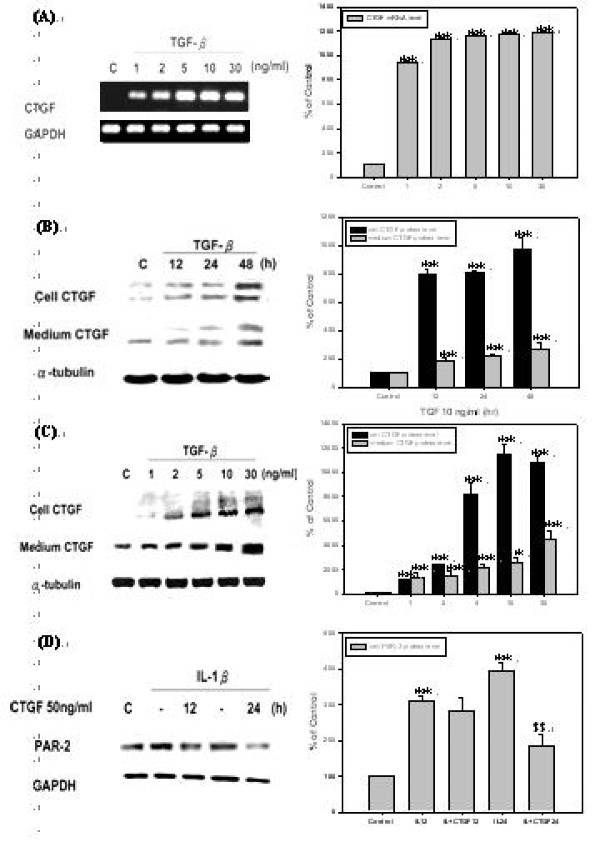
**TGF-β1 induces CTGF mRNA and protein expression level of *h*PSCs and CTGF suppresses PAR-2 protein level induced by IL-1β in *h*PSCs**. **(A) ***h*PSCs were incubated with 1, 2, 5, 10, and 30 ng/ml of TGF-β1 for 6 hours in serum-free DMEM following 24 hours of starvation (*n *= 3). Lane 1 as basal control and lane 2 to lane 6 were compared with basal control. **(B) ***h*PSCs were incubated in 10 ng/ml of TGF-β1 in serum-free DMEM for 12, 24, and 48 hours. TGF-β1 significantly induced cellular and medium CTGF protein level after 12, 24, and 48 hours (*n *= 3). Lane 1 as basal control and lane 2 to lane 4 were compared with basal control. **(C) ***h*PSCs were incubated with 1, 2, 5, 10, and 30 ng/ml of TGF-β1 for 24 hours in serum-free DMEM. TGF-β1 significantly induced cellular and medium CTGF protein level when treated with 1, 2, 5, 10, and 30 ng/ml of TGF-β1 (*n *= 3). Lane 1 as basal control and lane 2 to lane 6 were compared with basal control. **(D) ***h*PSCs were pre-treated with 10 ng/ml of IL-1β in serum-free DMEM for 30 minutes and then incubated in 50 ng/ml of CTGF for 12 and 24 hours in serum-free DMEM (*n *= 3). Lane 1 as basal control and lane 2 and lane 4 as positive controls of lane 3 and 5. Lane 2 and 4 were compared with basal control; lane 3 was compared with lane 2 and Lane 5 was compared with lane 4.

TGF-β1 was reported to be a strong inducer of CTGF in many cell types, such as fibroblast, hepatocytes, renal cells, and keratinocytes [20]. The effect of treating *h*PSCs with different dosage of TGF-β1 for 6 hrs on CTGF mRNA level was investigated. As shown by Fig. 4A, TGF-β1 induced CTGF mRNA expression following a dose-dependent pattern. Then treatments with 10 ng/ml of TGF-β1 for 12, 24, and 48 hrs or various concentration of TGF-β1 for 24 hrs were conducted, CTGF protein levels in both cellular and medium were determined. As shown by Fig. 4B, TGF-β1 induced CTGF production in a time-dependent manner after 12 hrs treatment and persisted to at least 48 hrs. TGF-β1 induced CTGF production also followed a dose-dependent manner after 24 hrs treatment as demonstrated by Fig. 4C.

### CTGF suppresses PAR-2 protein level induced by IL-1β in *h*PSCs

Since CTGF has been hypothesized as the major down-stream mediator of TGF-β1 [20], it was wondered if inhibition of PAR-2 expression by TGF-β1 in *h*PSCs not only via direct inhibition of p38 signal pathway, but also through the induction of CTGF which could further reduce PAR-2 protein level. The *h*PSCs were pre-treated with 10 ng/ml of IL-1β for 30 minutes, followed by treating with 50 ng/ml of CTGF for 12 and 24 hrs [21,22], after then cellular PAR-2 protein level was determined. As shown by Fig. 4D, CTGF repressed IL-1β-induced PAR-2 protein level after 24 hrs of treatment.

### CTGF inhibits IL-1β-mediated p38 MAPK activation, but activated ERK pathway of *h*PSCs

**Figure 5:**
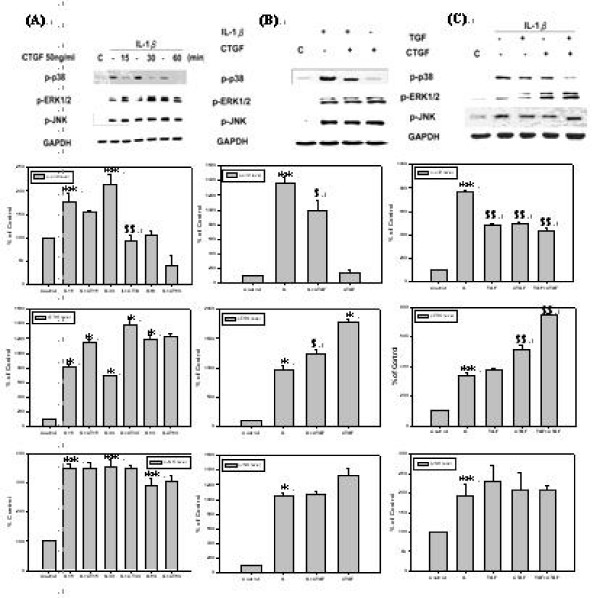
**CTGF inhibits IL-1β-mediated p38 MAPK activation, but activate ERK pathway of *h*PSCs**. **(A) ***h*PSCs were first incubated in serum-free DMEM for 24 hours and then pre-treated with 10 ng/ml of IL-1β in serum-free DMEM for 30 minutes and then incubated with 50 ng/ml of CTGF for 15, 30, and 60 minutes in serum-free DMEM (*n *= 3). Lane 1 as basal control and lane 2, 4, and 6 as positive controls of lane 3, 5, and 7. Lane 2, 4, and 6, were compared with basal control; lane 3 was compared with lane 2; Lane 5 was compared with lane 4; and lane 7 was compared with lane 6. **(B) ***h*PSCs were first incubated in serum-free DMEM for 24 hours and then pre-treated with 10 ng/ml of IL-1β in serum-free DMEM for 30 minutes and then incubated with 50 ng/ml of CTGF for 30 minutes in serum-free DMEM (*n *= 3). Lane 1 as basal control and lane 2 as positive control, lane 3 was compared with positive control and lane 4 was compared with basal control. **(C) ***h*PSCs were first incubated in serum-free DMEM for 24 hours and then pre-treated with 10 ng/ml of IL-1β in serum-free DMEM for 30 minutes and then treated with 10 ng/ml of TGF-β1 or 50 ng/ml of CTGF alone or combined for 30 minutes in serum-free DMEM (*n *= 3). Lane 1 as basal control and lane 2 as positive control, lane 3 to lane 5 was compared with positive control.

CTGF is an anabolic factor in OA process because this factor stimulates cell proliferation and ECM marker production, such as type II collagen and aggrecan in human chondrocytes [21,22]. CTGF was known as well to interact with TGF-β to deliver TGF-β to TGFRII [20], so it can enhance TGF-β activity. In light of this, it was thought that CTGF might enhance TGF-β-mediated p38 inhibition on the PAR-2 expression. To prove hypothesis, the *h*PSCs were treated with 50 ng/ml of CTGF for 15, 30, and 60 minutes following the exposure to 10 ng/ml of IL-1β, after then p-p38 level was determined. As shown by Fig. 5, CTGF inhibited IL-1β induced p-p38 level but not p-ERK or p-JNK level after 30 minutes of treatment (Fig. 5A), while treatment with CTGF alone does not affect p-p38 level of *h*PSCs (Fig. 5B). In order to validate that TGF-β inhibits p-p38 is mediated by CTGF-TGF-β interaction, p-p38 inhibition effect among treatments with TGF-β1, CTGF, and combination of both were compared. As shown by Fig. 5C, when combined treatment with both of TGF-β1 and CTGF demonstrates a greater p-p38 inhibition effect than that treated with TGF-β1 or CTGF alone.

### TGF-β1 up-regulates mRNA and protein level of TIMP-3 in *h*PSCs

**Figure 6:**
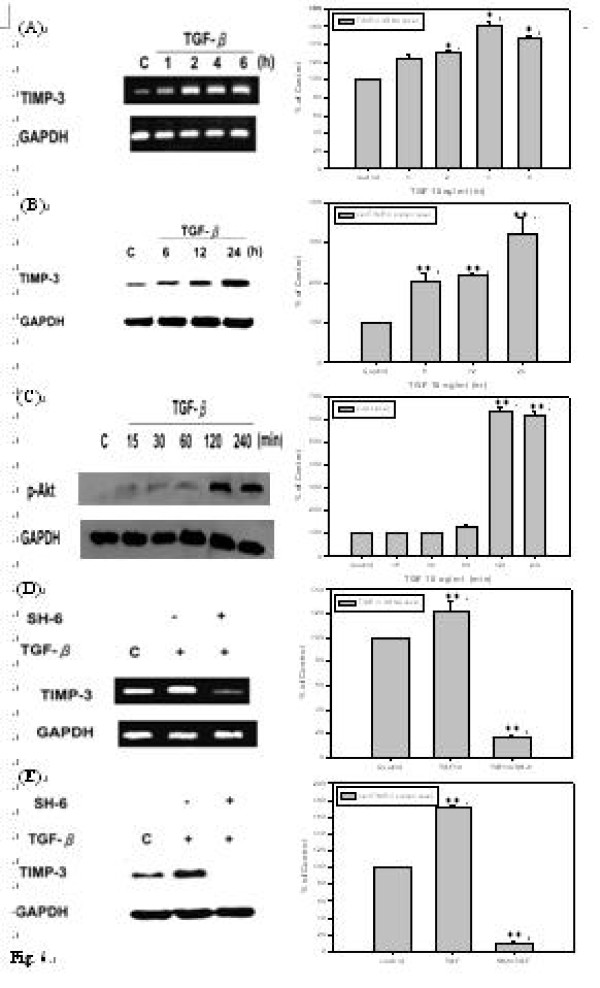
**TGF-β1 up-regulates mRNA and protein level of TIMP-3 in *h*PSCs and TGF-β1 induces TIMP-3 expression via Akt pathway in *h*PSCs**. **(A) ***h*PSCs were incubated with 10 ng/ml of TGF-β1 in serum-free DMEM for 1, 2, 4, and 6 hours following starvation for 24 hours. TIMP-3 mRNA was significantly up-regulated after 1, 2, 4, and 6 hours and had the maximal effect in the time of 4th hour (*n *= 3). Lane 1 as basal control and lane 2 to lane 5 were compared with basal control. **(B) ***h*PSCs were incubated with 10 ng/ml of TGF-β1 in serum-free DMEM for 6, 12, and 24 hours. TIMP-3 protein was significantly induced after 6, 12, and 24 hours (*n *= 3). Lane 1 as basal control and lane 2 to lane 4 were compared with basal control. **(C) ***h*PSCs were incubated in starvation medium for 24 hours and then treated with 10 ng/ml of TGF-β1 in serum-free DMEM for 15, 30, 60, 120, and 240 minutes (*n *= 3). Lane 1 as basal control and lane 2 to lane 6 were compared with basal control. **(D) ***h*PSCs were incubated in starvation medium for 24 hours and then treated with 10 μM of SH-6 for 30 minutes followed by incubated with 10 ng/ml of TGF-β1 in serum-free DMEM for 4 hours (*n *= 3). Lane 1 as basal control and lane 2 as positive control, lane 3 was compared with positive control. **(E) ***h*PSCs were treated with 10 μM of SH-6 for 30 minutes followed by incubated with 10 ng/ml of TGF-β1 in serum-free DMEM for 24 hours (*n *= 3). Lane 1 as basal control and lane 2 as positive control, lane 3 was compared with positive control.

It was reported that TGF-β1 can reduce MMPs level in human chondrocytes due to its ability to induce TIMP-3, a nature inhibitor of MMPs *in vivo *[23]. Therefore, whether or not TGF-β can induce TIMP-3 production in *h*PSCs was investigated. After treatment with 10 ng/ml of TGF-β1 in a time course, the protein and mRNA levels of TIMP-3 in the *h*PSCs were determined. As shown by Fig. 6, TIMP-3 mRNA level was induced by TGF-β1 after 1 hr treatment and had greater effect at the time point of 4 hr (Fig. 6A). TGF-β1 also induced TIMP-3 protein expression followed a time-dependent manner during 6 to 24 hrs after treatment (Fig. 6B).

### TGF-β1 induces TIMP-3 expression via Akt pathway in *h*PSCs

It has been reported that TGF-β1 induced TIMP-3 expression through Akt pathway in human chondrocytes [23]. If this induction through the same pathway in *h*PSCs was then determined. *h*PSCs were treated with 10 ng/ml of TGF-β1 for various time periods from 15 to 240 minutes and then p-Akt level was analyzed. As shown by Fig. 6, TGF-β1 induced p-Akt level after 120 minutes of treatment and this induction persisted at least 240 minutes (Fig. 6C). To further confirm it, the cells were pre-treated with SH-6, an inhibitor of Akt, for 30 minutes, and then 10 ng/ml of TGF-β1 was added and TIMP-3 mRNA and protein level were analyzed at the time point of 4 hr and 24 hr. As shown by Figs. 6D and 6E, SH-6 almost blocked total TGF-β1-induced TIMP-3 mRNA and protein expression, respectively.

### TIMP-3 blocks MMP-13 activity in culture medium of *h*PSCs

**Figure 7:**
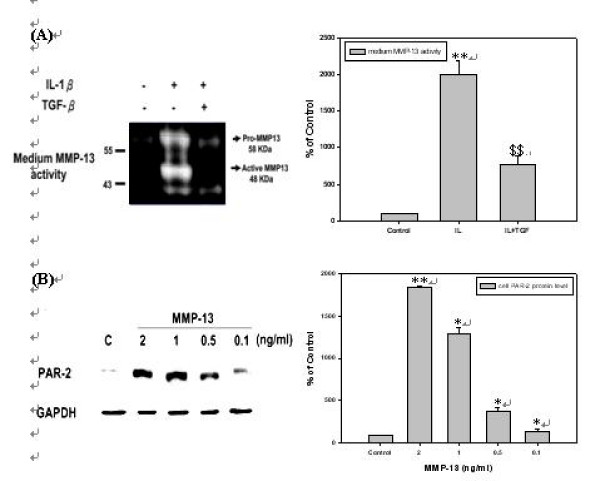
**TIMP-3 blocks MMP-13 activity and blocking of MMP-13 function results in down-regulation of PAR-2 protein level of *h*PSCs**. **(A) ***h*PSCs were incubated with 10 ng/ml of IL-1β in serum-free DMEM for 12 hours and then co-incubated with 10 ng/ml of TGF-β1 in serum-free DMEM for 12 hours (*n *= 3). Lane 1 as basal control and lane 2 as positive control, lane 3 was compared with positive control. **(B) ***h*PSCs were incubated with 2, 1, 0.5, and 0.1 ng/ml of MMP-13 in serum-free DMEM for 24 hours (*n *= 3). Lane 1 as basal control and lane 2 to lane 5 were compared with basal control.

It is known that TIMP-3 inhibits MMP-13 by competing the catalytic pocket of MMP-13 with its substrate, collagen. MMP-13 activity was examined in culture medium of *h*PSCs to confirm that TIMP-3 functioned. The cells were pre-treated with 10 ng/ml of IL-1β for 12 hrs to induce MMP-13 secretion, and then those were co-treated with 10 ng/ml of TGF-β1 for 12 hrs to induce TIMP-3 production and then MMP-13 activity was investigated in conditioned medium by collagen zymography. As shown by Fig. 7A, MMP-13 activity was significantly induced by IL-1β, but when the cells were treated with TGF-β1, TIMP-3 was produced and subsequently MMP-13 activity was blocked.

### Blocking of MMP-13 function resulted in down-regulation of PAR-2 protein level of *h*PSCs

It was observed that suppression of MMP-13 activity also reduced PAR-2 protein level in human chondrocytes. It was because that inhibition of MMP-13 could slow down cartilage break and then reducing stimulating factor for proinflammatory cytokines releasing. As a result, inhibition of MMP-13 function leads to reduce PAR-2 expression indirectly. Based on the reported study that concentration of MMP-13 in synovial fluid of OA patients lies in the range of 0.1~1.5 ng/ml [24], *h*PSCs were treated with various concentrations of MMP-13 in this range for 24 hrs and then cellular PAR-2 protein level was determined. As shown by Fig. 7B, the cells treated with the higher concentration of MMP-13, the higher protein level of PAR-2 was expressed, but vice versa.

## Discussion

This study is the first report to demonstrate the pathway how PAR-2 is repressed by TGF-β in *h*PSCs. According to available reports [2], we know that PAR-2 is unique in its distribution property because PAR-2 is the only member of this family expressed in human chondrocytes. In contrast, *h*PSCs express PAR-1, PAR-2, and PAR-3. But in our study, it was observed that PAR-1, PAR-2, and PAR-3 are expressed in both human chondrocytes and *h*PSCs. Nevertheless, we found that the mRNA levels of PAR-1, PAR-2, and PAR-3 were significantly induced by IL-1β in human chondrocytes, but only PAR-2 mRNA was up-regulated by IL-1β in *h*PSCs. In addition, PAR-2 had higher expression level in *h*PSCs than that in human chondrocytes, which suggests that PAR-2 expression level in *h*PSCs might play a more significant role in the progression of OA.

PAR-1 is related with chemokine release, while PAR-2 is directly related with inflammation [18]. It is possible that during early phase of OA, IL-1β stimulates PAR-2 expressing by *h*PSCs via autocrine pathway and results more IL-1β being secreted to up-regulate PAR-1 of human chondrocytes and stimulating chemokine such as IL-6, IL-8, eotaxin and MCP-1 releasing by human chondrocytes that enhances OA severity.

In *h*PSCs, IL-1β up-regulated PAR-2 gene expression in short time (Fig. 1B, mRNA level), suggesting that PAR-2 expression mainly occurs in the early phase of acute inflammation. However, induction of PAR-2 protein level by IL-1β was from 12 to 48 hrs in *h*PSCs (Fig. 1B, protein level), suggesting that PAR2-mediated inflammatory response in OA synovial cells through an amplification effect.

Results showed that TGF-β inhibited PAR-2 protein level induced by IL-1β followed a time dependent manner (Fig. 2C). This inhibition effect persisted for at least 48 hrs, suggesting that TGF-β might inhibit IL-1β induced PAR-2 through multiple pathways. It was proved in this study that TGF-β inhibits PAR-2 expression in *h*PSCs through three pathways: by directly inhibition on p38 phosphorylation, by inducing TIMP-3 which in turn results MMP-13 dysfunction to ease inflammation cascade, and by induction CTGF to block p38 signal pathway leading to inhibit the proliferation of inflammatory cells.

It was seen that TGF-β inhibited p-p38 pathway, the major signal pathway which mediated PAR-2 expression by *h*PSCs (Fig. 2A), soon after 15 minutes of treatment (Fig. 3). This is the first mechanism of TGF-β suppressed PAR-2 which occurred at the time point of 12 to 24 hrs as observed by Fig. 2C.

It should be noticed that PAR-2 was up-regulated under the exposure to higher MMP-13 concentration for 24 hrs in *h*PSCs (Fig. 7B). This might be due to higher MMP-13 concentration further enhanced inflammatory response of synovial cells to stimulate IL-1β secretion. It was considered that up-regulation of PAR-2 by MMPs might be indirectly. Hence, blocking MMP-13 can also prevent over-expression of PAR-2. Besides direct inhibition of p38 signal pathway, TGF-β might inhibit PAR-2 through TGF-β-TIMP-MMP-PAR2 cascade. According to our studies, TIMP-3 protein was significantly induced by TGF-β after 6 to 24 hrs of treatment (Fig. 6B) to block MMP-13. Once MMP-13 was blocked, PAR-2 level decreased within 24 hrs (Fig. 7B). In brief, inhibition of PAR-2 expression by TGFβ-TIMP-MMP-PAR2 cascade lies during the time period of 24 to 36 hr.

It was observed that CTGF activated ERK but inhibited p38 pathway induced by IL-1β (Fig. 5A and Fig. 5B) in *h*PSCs, of which was different for CTGF in human chondrocytes which activated both ERK and p38 pathway [20]. As reported, CTGF stimulates proliferation via p38 MAPK resulting in chondrocytes growth and differentiation through p42/p44 MAPK pathway resulting in ECM specific marker expression. In contrast, CTGF activated p42/p44 rather than p38 in *h*PSCs, suggesting that CTGF mediates differentiation and ECM specific marker expression (data not shown) via p42/p44 pathway but not via p38 pathway (Fig. 5A and Fig. 5B). This might be the reason explained that over-expression of CTGF in human chondrocytes results in osteophyte formation but over-expression of CTGF in *h*PSCs does not lead to osteophyte formation.

It is noticeable that CTGF enhanced TGF-β-mediated p38 inhibition in *h*PSC (Fig. 5C). This suggests that TGF-β inhibits p38 through CTGF and CTGF enhances TGF-β activity by presenting TGF-β to TGFR [20]. So, another pathway that TGF-β inhibits p38 is by inducing CTGF to reduce phospho-p38 level. It was observed that TGF-β significantly induced CTGF protein level after 12 to 48 hrs (Fig. 4B) and then PAR-2 was repressed by CTGF after 24 hrs (Fig. 4D). It was concluded that the inhibition of p38 induced by IL-1β via TGF-β-CTGF-p38 cascade occurs during the time period of 36 to 48 hr.

In conclusion, this study suggests that TGF-β inhibits IL-1β induced PAR-2 expression level through three pathway at various time points. First, TGF-β directly inhibits IL-1β induced p-p38 level within 24 hrs. Secondary, TGF-β induces TIMP-3 expression resulting in suppression of MMP-13 function, leading that PAR-2 over-expression being eased off by TGF-β-TIMP3-MMP13 cascade occurs during the time period of 24 to 36 hr. Finally, TGF-β induces CTGF formation after 12 hrs, and CTGF reduces PAR-2 protein level in *h*PSCs after 24 hrs, this TGF-β-CTGF-PAR2 cascade occurs during the time period of 36 to 48 hr. Because IL-1β-induced PAR-2 expressed in *h*PSCs might play a significantly important role in early phase of OA, PAR2 repression by exogenous TGF-β or other agents might be an ideal therapeutic target to prevent OA from progression.

## Abbreviations

ADAM: a disintegrin and metalloproteinase-like (or, alternatively, adamalysin); ADAMTS: a disintegrin and metalloproteinase with thrombo spondin motifs (or, alternatively, adamalysin-thrombospondin); ECM: extracellular matrix; GAG: glycosaminoglycan; IL: interleukin; MMPs: matrix metalloproteinases; TIMP: tissue inhibitor of metalloproteinase; PAR-2: proteinase-activated receptor-2; TGF-β: Transforming growth factor-β; CTGF: connective tissue growth factor; TNF-α: Tissue necrotic factor-α; BMPs: bone morphogenetic proteins; PI3K/Akt: Phosphoinositide 3-kinase/Akt; p42/p44 MAPK: also called Erk2 and Erk1.

## Competing interests

The authors declare that they have no competing interests.

## Authors' contributions

SH, YC and SS conceived of the study, and participated in its design and coordination. MT designed research; HT performed research; HT, MT and CH analyzed data and wrote the paper. All authors read and approved the final manuscript.
